# Artificial intelligence in COPD CT images: identification, staging, and quantitation

**DOI:** 10.1186/s12931-024-02913-z

**Published:** 2024-08-22

**Authors:** Yanan Wu, Shuyue Xia, Zhenyu Liang, Rongchang Chen, Shouliang Qi

**Affiliations:** 1 College of Medicine and Biological Information Engineering, Northeastern University, Shenyang, China; 2 Key Laboratory of Intelligent Computing in Medical Image, Ministry of Education, Northeastern University, Shenyang, China; 3 Respiratory Department, Central Hospital Affiliated to Shenyang Medical College, Shenyang, China; 4 Key Laboratory of Medicine and Engineering for Chronic Obstructive Pulmonary Disease in Liaoning Province, Shenyang, China; 5 State Key Laboratory of Respiratory Disease, National Clinical Research Center for Respiratory Disease, Guangzhou Institute of Respiratory Health, The National Center for Respiratory Medicine, The First Affiliated Hospital of Guangzhou Medical University, Guangzhou, China; 6 Shenzhen Institute of Respiratory Disease, Shenzhen People’s Hospital, Shenzhen, China

**Keywords:** Chronic obstructive pulmonary disease, Artificial intelligence, Computed tomography

## Abstract

Chronic obstructive pulmonary disease (COPD) stands as a significant global health challenge, with its intricate pathophysiological manifestations often demanding advanced diagnostic strategies. The recent applications of artificial intelligence (AI) within the realm of medical imaging, especially in computed tomography, present a promising avenue for transformative changes in COPD diagnosis and management. This review delves deep into the capabilities and advancements of AI, particularly focusing on machine learning and deep learning, and their applications in COPD identification, staging, and imaging phenotypes. Emphasis is laid on the AI-powered insights into emphysema, airway dynamics, and vascular structures. The challenges linked with data intricacies and the integration of AI in the clinical landscape are discussed. Lastly, the review casts a forward-looking perspective, highlighting emerging innovations in AI for COPD imaging and the potential of interdisciplinary collaborations, hinting at a future where AI doesn’t just support but pioneers breakthroughs in COPD care. Through this review, we aim to provide a comprehensive understanding of the current state and future potential of AI in shaping the landscape of COPD diagnosis and management.

## Background

### The prevalence and impact of COPD

Chronic obstructive pulmonary disease (COPD) remains one of the predominant public health challenges of the 21st century. With a global footprint spanning diverse demographic and geographic settings, COPD presents a multifaceted clinical picture marked by persistent respiratory symptoms and airflow limitations due to airway and alveolar abnormalities [1]. Primarily driven by prolonged exposure to noxious particles or gases, especially those originating from tobacco smoking, the pathological underpinnings of COPD are complex and varied, involving chronic inflammation, structural changes, and repair processes that affect both the larger airways and the peripheral lung [2].

As of the latest global estimates, over 250 million people suffer from COPD worldwide, making it the third leading cause of death by 2030 [3]. Economically, the disease also places a heavy toll on healthcare systems, with direct medical costs and productivity losses amounting to billions annually [4]. These alarming statistics underline the pressing need for early identification, accurate staging, and effective management strategies.

This review aims to journey through the convergence of COPD imaging from computed tomography (CT) with the capabilities of AI, underscoring the current achievements, challenges, and the road ahead.

### The power of CT imaging in COPD

Medical imaging has proven indispensable in the landscape of respiratory medicine [5]. CT imaging, with its high resolution and ability to visualize lung structures in detail, has provided a platform for in-depth investigations into the complex manifestations of COPD [6, 7].

Unlike spirometry, which gives a global measure of lung function, CT imaging can localize and characterize the pathological abnormalities of COPD [8]. It illuminates the heterogeneity inherent in the disease-whether it be the bullous formations of emphysema, thickening of the bronchial walls, or alterations in the pulmonary vessels [2, 6, 9, 10]. CT imaging is crucial not just for diagnosis but also for tailoring patient-specific interventions, monitoring disease progression, and evaluating therapeutic efficacy [11].

Further, staging of COPD, which is vital for prognostication and management decisions, has traditionally leaned heavily on physiological parameters. However, the staging paradigm is experiencing a shift. With the advent of quantitative CT techniques, objective measurements related to airway thickness, lung volume, and parenchymal attenuation are becoming part of the COPD assessment lexicon [12, 13]. These metrics provide a more nuanced understanding of disease severity and its spatial distribution within the lungs.

As COPD comprises heterogeneous imaging phenotypes, including emphysema, airway changes, and vessel modifications, CT imaging offers a non-invasive window into the structural abnormalities that define these phenotypes [6, 14, 15]. With the surge in technological advancements, particularly the integration of artificial intelligence (AI) in medical imaging, the landscape of COPD diagnosis and management is set for a transformative shift [16, 17].

Yet, the vast potential of CT imaging is not without its challenges. The sheer volume of data from high-resolution scans demands intensive manual scrutiny, making the interpretation time-consuming and prone to variability [18]. Herein lies the promise of AI. By harnessing algorithms trained on vast datasets, AI can automate, augment, and refine the image analysis process, bringing precision and consistency to the forefront [19–21].

## AI in medical imaging: an overview

### Machine learning vs. deep learning: differentiating the two foundational approaches

AI encompasses a spectrum of techniques aimed at simulating human-like intelligence. Two standout sub-domains are machine learning (ML) and deep learning (DL), each having distinct attributes and applications in medical imaging.

ML employs algorithms that learn from data to make predictions. In medical imaging, these algorithms often use labeled datasets to discern patterns and offer diagnostics [22, 23]. Various types include: supervised learning: algorithms learn from labeled data, aiding in tasks like disease subtype classification [24] and treatment response prediction [25]. Unsupervised learning: patterns are identified without labeled data, useful for segmenting similar regions in CT images [26].

DL, an advanced subset of ML, operates on multi-layered neural networks. The star of DL in medical imaging is the convolutional neural network (CNN), optimized for image processing by detecting features ranging from basic edges to complex patterns [27]. Its prowess in COPD imaging stems from its ability to discern intricate image features, offering detailed classifications [28]. A trinity of factors supports DL’s ascendancy in imaging: extensive labeled datasets, sophisticated network designs, and burgeoning computational power [29].

**Fig. 1:**
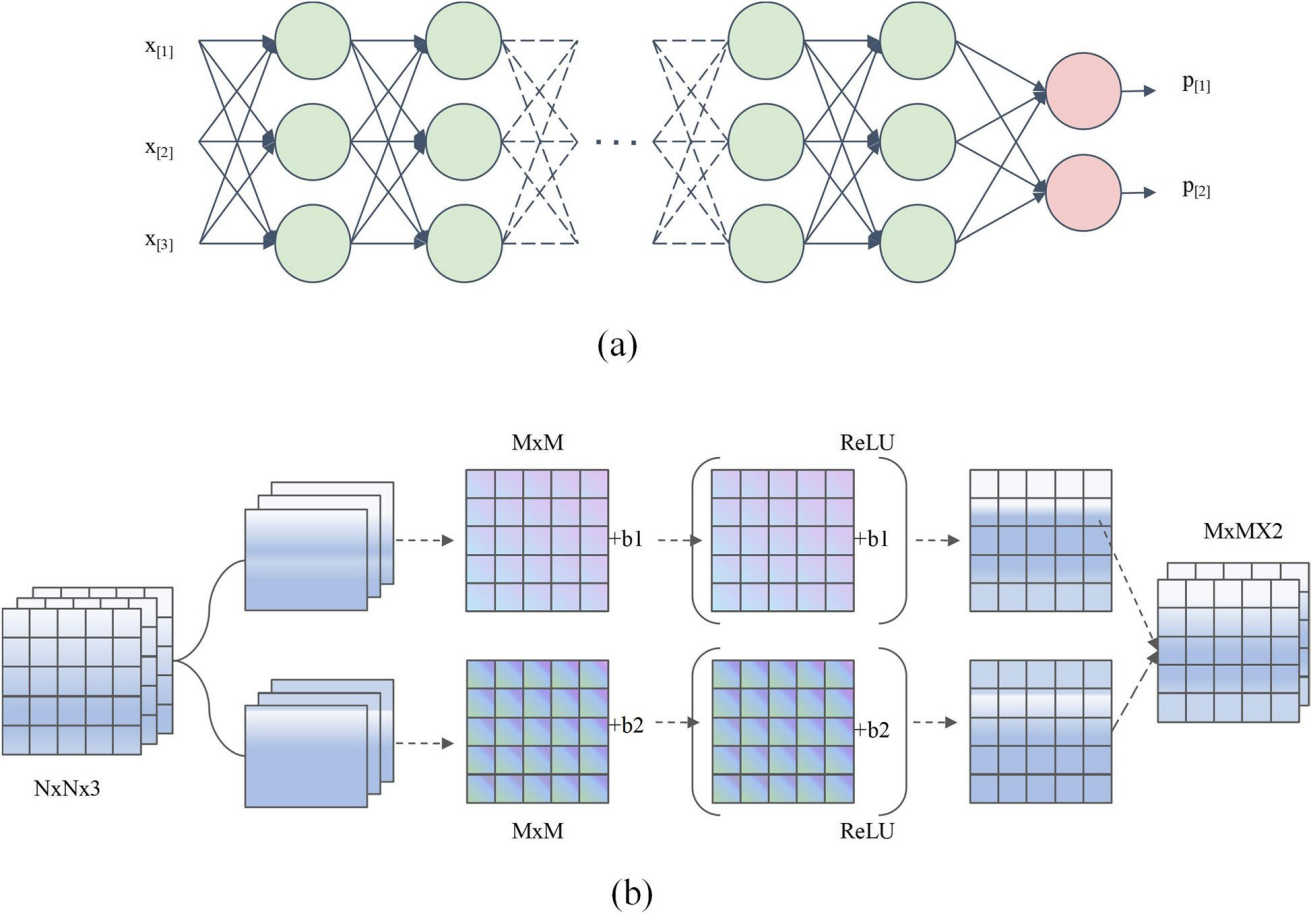
Fundamental concepts of deep learning. **a** MLP Prediction with 2 outputs. **b** Convolutional operation

Figure 1 illustrates two fundamental concepts in deep learning: the multi-layer perceptron (MLP) and convolutional operations. Figure 1a depicts a multi-layer perceptron, which is a type of feedforward artificial neural network. The MLP consists of an input layer, one or more hidden layers, and an output layer. In this specific example, the input layer comprises three variables (or features), denoted as x1, x2, and x3. These input variables are fed into the hidden layers, where each neuron applies a nonlinear activation function to the weighted sum of its inputs. The activation function introduces non-linearity into the network, enabling it to learn complex mappings between the input and output spaces. The output layer of the MLP produces two values, representing the probabilities of the input belonging to two different classes, denoted as p1 and p2. The MLP learns to classify the input data by adjusting the weights of the connections between neurons during the training process, typically using optimization algorithms such as stochastic gradient descent.

Figure 1b illustrates the process of convolutional operations, which are fundamental building blocks in CNN. CNN is particularly effective in processing grid-like data, such as images or time series. In this example, the input is a three-channel tensor, which can be thought of as an RGB image. The convolutional operation involves sliding a set of learnable filters (or kernels) over the input tensor, performing element-wise multiplications, and summing up the results to produce a feature map. The figure shows two convolutional kernels being applied to the input tensor, resulting in two output channels. Each kernel has a specific set of weights that are learned during the training process to extract relevant features from the input. The convolutional operation exploits spatial locality and parameter sharing, enabling the network to learn translation-invariant features and capture local patterns in the data. By stacking multiple convolutional layers, CNNs can learn hierarchical representations, with lower layers capturing low-level features (e.g., edges and textures) and higher layers capturing more abstract and semantic information.

The combination of MLPs and convolutional operations forms the backbone of many deep learning architectures. MLPs are commonly used in the final stages of CNNs for classification or regression tasks, while convolutional layers are employed to extract spatial features from the input data. By leveraging the power of deep neural networks and convolutional operations, deep learning has achieved remarkable success in various domains, including computer vision [30], natural language processing [31], and speech recognition. The ability to automatically learn hierarchical representations from raw data has revolutionized the field of artificial intelligence and opened up new possibilities for solving complex problems.

**Fig. 2:**
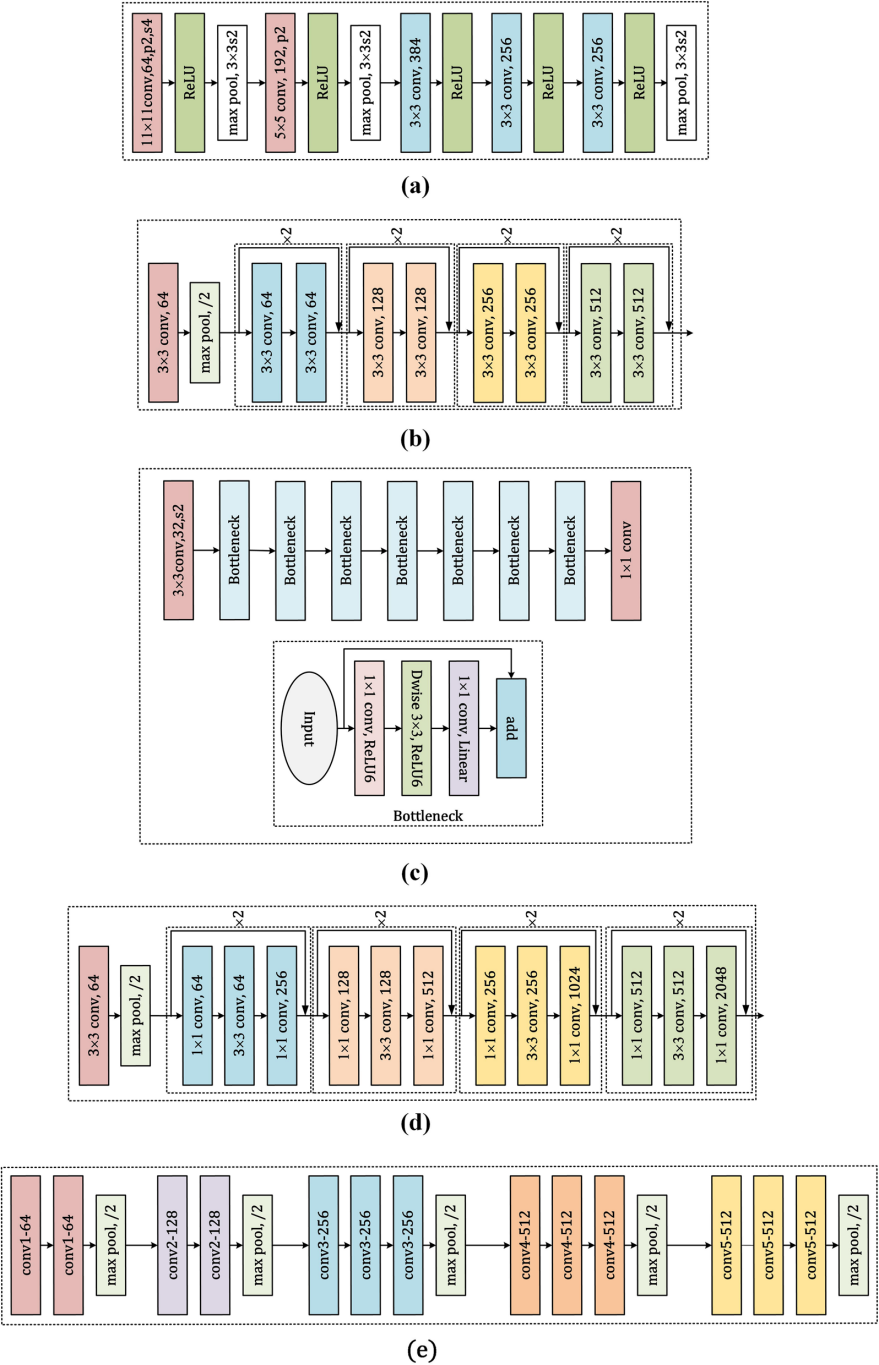
Some main CNN architectures referring to [32]. **a** AlexNet, **b** ResNet18, **c** Mobilenet-v2, **d** ResNet26, and **e** VGG16

Figure 2 presents an overview of several influential CNN architectures. The figures are referred to the previous study [32]: (a) AlexNet: Developed by Krizhevsky et al. in 2012 [33], AlexNet is a pioneering CNN architecture that achieved remarkable performance on the ImageNet classification task. It consists of five convolutional layers followed by three fully connected layers. AlexNet introduced the use of rectified linear unit (ReLU) activation functions and employed techniques such as data augmentation and dropout regularization to improve generalization. (b) ResNet18: ResNet, short for Residual Network, is a family of CNN architectures introduced by He et al. in 2015 [30]. ResNet18 is a specific instance of the ResNet architecture with 18 layers. The key innovation of ResNet is the introduction of residual connections, which allow the network to learn residual functions with reference to the input layer, enabling the training of much deeper networks without the vanishing gradient problem. (c) MobileNet-v2: MobileNet is a family of efficient CNN architectures designed for mobile and embedded vision applications. MobileNet-v2, proposed by Sandler et al. in 2018 [34], builds upon the ideas of depthwise separable convolutions and introduces inverted residual connections. This architecture achieves a good balance between accuracy and computational efficiency, making it suitable for resource-constrained devices. (d) ResNet26: ResNet26 is another variant of the ResNet architecture, similar to ResNet18 but with 26 layers [35]. It follows the same principles of residual learning, allowing for the training of deeper networks while mitigating the vanishing gradient problem. ResNet26 offers a trade-off between network depth and computational complexity. (e) VGG16: VGG16, introduced by Simonyan and Zisserman in 2014 [36], is a CNN architecture known for its simplicity and effectiveness. It consists of 16 layers, including 13 convolutional layers and 3 fully connected layers. VGG16 uses small convolutional filters (3x3) and employs a uniform architecture, making it easy to understand and implement. Despite its depth, VGG16 has been widely adopted and has served as a foundation for many subsequent CNN architectures.

These CNN architectures have been instrumental in advancing the field of deep learning and have been applied to various computer vision tasks, including image classification, object detection, and semantic segmentation. Each architecture has its own unique characteristics and trade-offs in terms of accuracy, computational complexity, and memory requirements. Understanding the design principles and performance characteristics of these architectures is crucial for selecting the appropriate model for a given task and for developing new CNN architectures tailored to specific domain requirements, such as medical image analysis for the characterization and recognition of COPD using CT scans [37, 38].

In sum, while ML and DL both offer value in medical imaging, the selection between them hinges on the specific demands of the task at hand. Their differentiated roles will be further proved as we delve deeper into AI’s integration with COPD imaging.

### Applications of AI in medical imaging

Radiology, an indispensable branch of medical diagnostics, has always been at the forefront of technological integration. The recent embrace of AI within radiology signifies a paradigm shift, augmenting the accuracy, efficiency, and capabilities of imaging modalities. (1) Image analysis and interpretation: AI-powered tools have streamlined image analysis, enhancing detection sensitivity and reducing manual errors. For instance, algorithms can assist radiologists in identifying early signs of pathologies like tumors, vascular anomalies, or pulmonary conditions that might be subtle or ambiguous in initial scans [39–42]. (2) Workflow optimization: Beyond image interpretation, AI facilitates streamlined workflows in radiological settings. Tools can prioritize reading lists based on urgency, predict no-show appointments, or automate documentation processes, thus improving clinical efficiency [43, 44]. (3) Radiation dose reduction: One of the concerns in radiological procedures is the radiation dose. AI algorithms can reconstruct high-quality images from lower-dose scans, striking a balance between image clarity and patient safety [45, 46]. (4) Predictive analysis: AI’s ability to integrate imaging data with electronic health records allows for predictive modeling. This offers insights into potential disease trajectories, response to treatments, or even risks of complications, enabling personalized patient care [47, 48]. (5) Advanced imaging techniques: AI augments traditional imaging modalities like MRI or CT with advanced techniques. For instance, AI-powered synthetic MRI can generate multiple image sequences from a single acquisition, reducing scan times [49–51]. As radiology continues its evolutionary journey, AI stands as a beacon, promising transformative changes. Its integration not only amplifies diagnostic precision but also heralds a more patient-centric approach, where tailored interventions and enhanced safety become the norm.

According to our previous studies [32, 52, 53], the following will provide three examples of the application of AI, especially deep learning methods, to lung CT images.

**Fig. 3:**
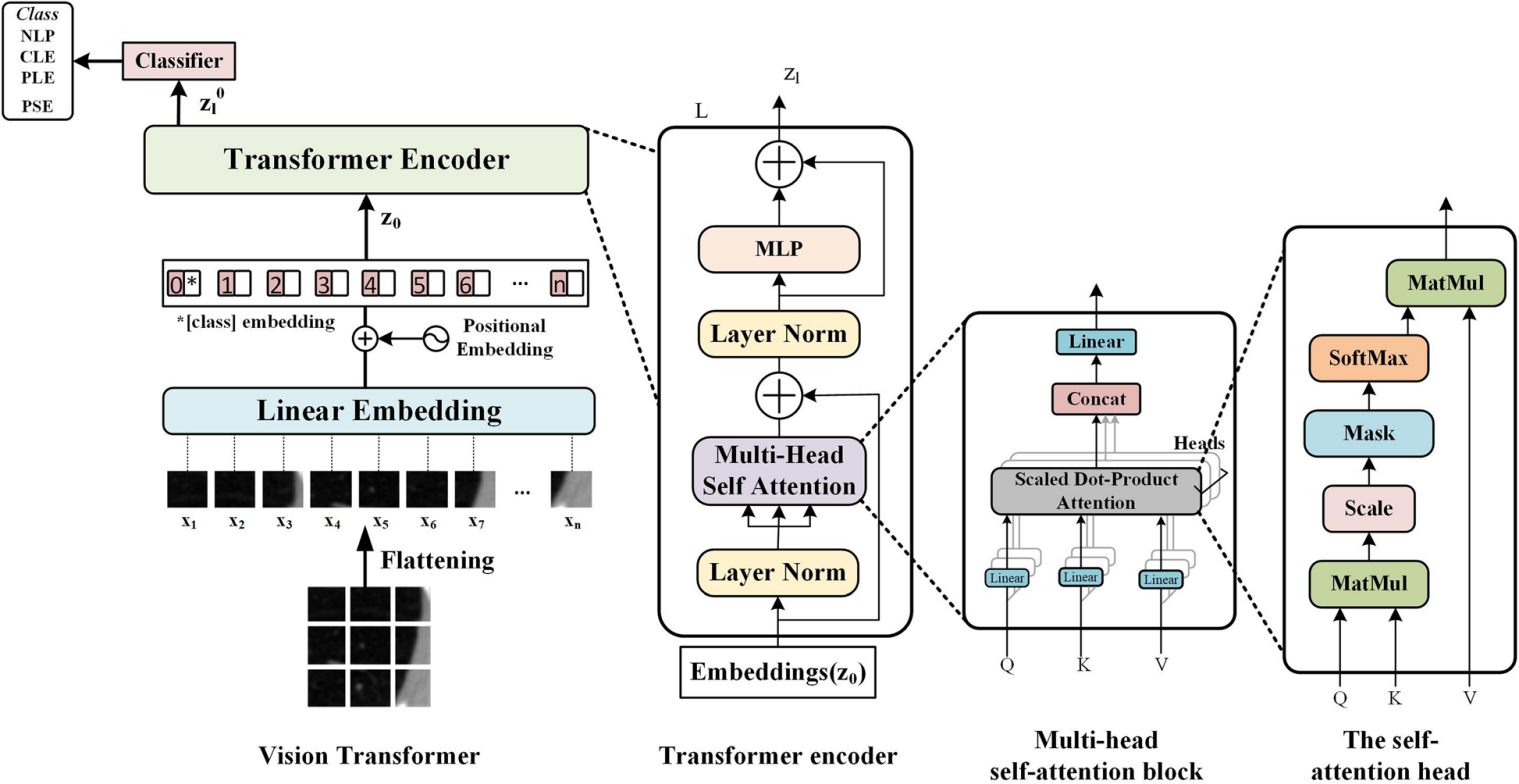
The architecture of Vision Transformer and its application in emphysema subytpe classification [52]

Figure 3 introduces the Vision Transformer (ViT) and its application in emphysema subtype classification. The ViT model, introduced by Dosovitskiy et al. in 2020 [54], adapts the Transformer architecture, originally designed for natural language processing, to the task of image classification. In this specific application, the ViT model is employed to classify emphysema subtypes, which are important indicators of COPD severity and progression.

The input to the ViT model is a large patch of slice in CT scan, which is preprocessed and divided into a sequence of fixed-size patches. These patches are linearly embedded and augmented with positional embeddings to preserve spatial information. The embedded patches are then fed into the Transformer encoder, which consists of multiple layers of multi-head self-attention and feed-forward networks. The self-attention mechanism allows the model to capture long-range dependencies and learn global context, enabling it to effectively capture the spatial patterns and textural characteristics of emphysema subtypes.

The output of the Transformer encoder is a sequence of feature vectors, which are then aggregated using a classification token and passed through a MLP to obtain the final emphysema subtype predictions. The ViT model is trained on a labeled dataset of CT scans with annotated emphysema subtypes, using techniques such as data augmentation and transfer learning to improve generalization and performance.

The application of ViT for emphysema subtype classification offers several advantages. First, the self-attention mechanism enables the model to capture long-range dependencies and global context, which is crucial for accurately identifying the spatial patterns and textural characteristics of different emphysema subtypes. Second, the ViT architecture is highly scalable and can be trained on large datasets, for example, ImageNet, allowing for the learning of rich and expressive feature representations. Finally, the ViT model has shown promising results in various medical image analysis tasks, demonstrating its potential for improving the accuracy and efficiency of emphysema subtype classification in COPD assessment.

**Fig. 4:**
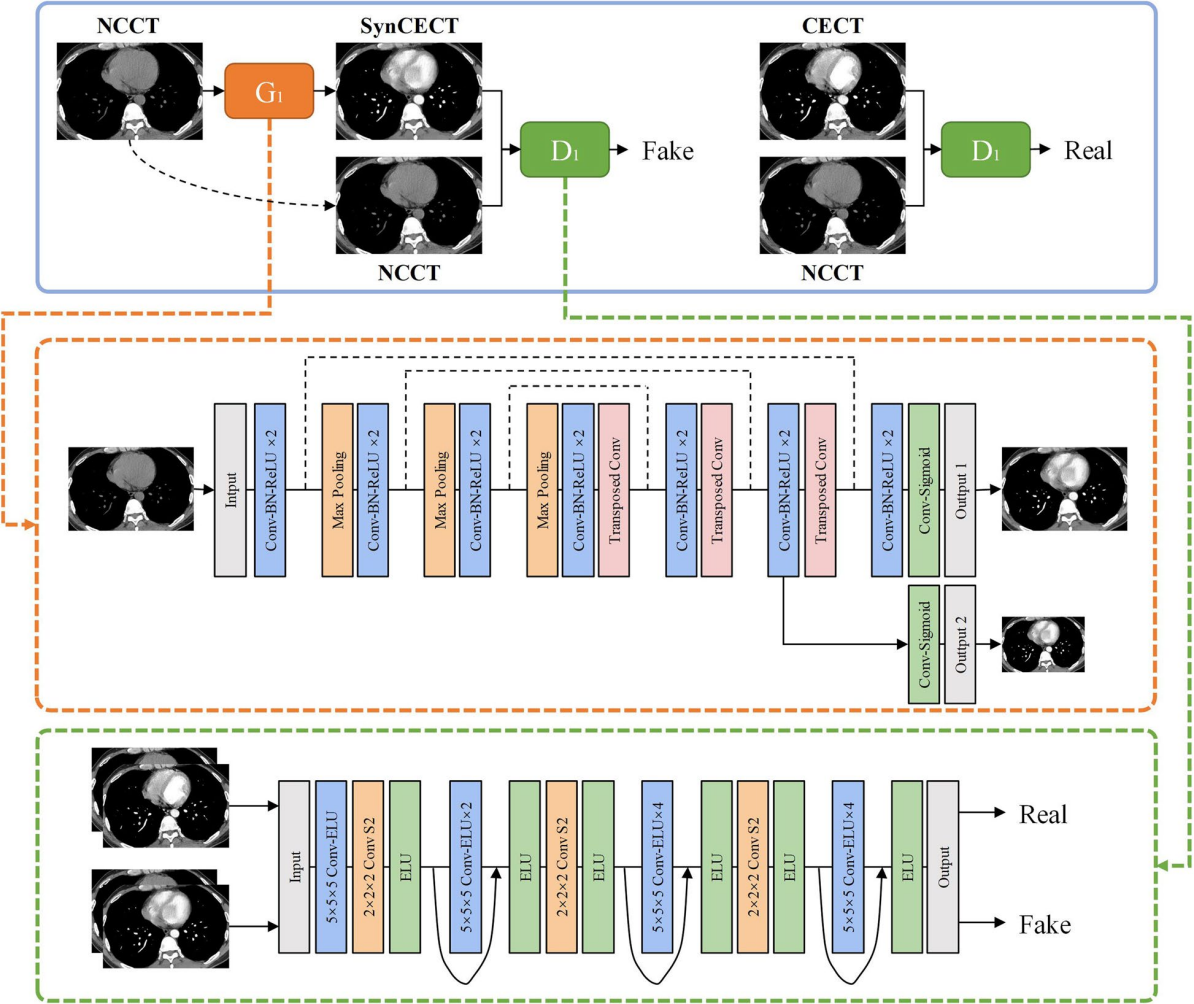
The generative adversarial network (GAN) for synthesizing contrasted-enhanced or non-contrasted CT [53]. The proposed Synthesizer based on GAN and consists the generator and the discriminator

Moreover, as depicted in Fig. 4, The generative adversarial network (GAN) related method was proposed for synthesizing contrasted-enhanced or non-contrasted CT. The proposed synthesizer’s network architecture comprises a generator and a discriminator. The generator’s purpose is to create synthetic images, while the discriminator’s role is to differentiate between authentic and generated images, enabling the generator to learn to produce realistic contrasted enhanced (CE) CT or Non-contrasted (NC) CT images.

The NC CT is input into generator (G1) to generate SynCECT. The NC CT and SynCECT are then concatenated along the channel dimension and fed into the discriminator (D1). The discriminator generates a probability map indicating whether the input image is a SynCECT or a real CE CT. Additionally, the NC CT and real CE CT are combined and input into the discriminator to produce a probability map. The generator and discriminator continue to compete until an equilibrium is achieved. The backbone of the generator (Fig. 4) is based on the 3D U-Net architecture, which consists of an encoder and a decoder section.

**Fig. 5:**
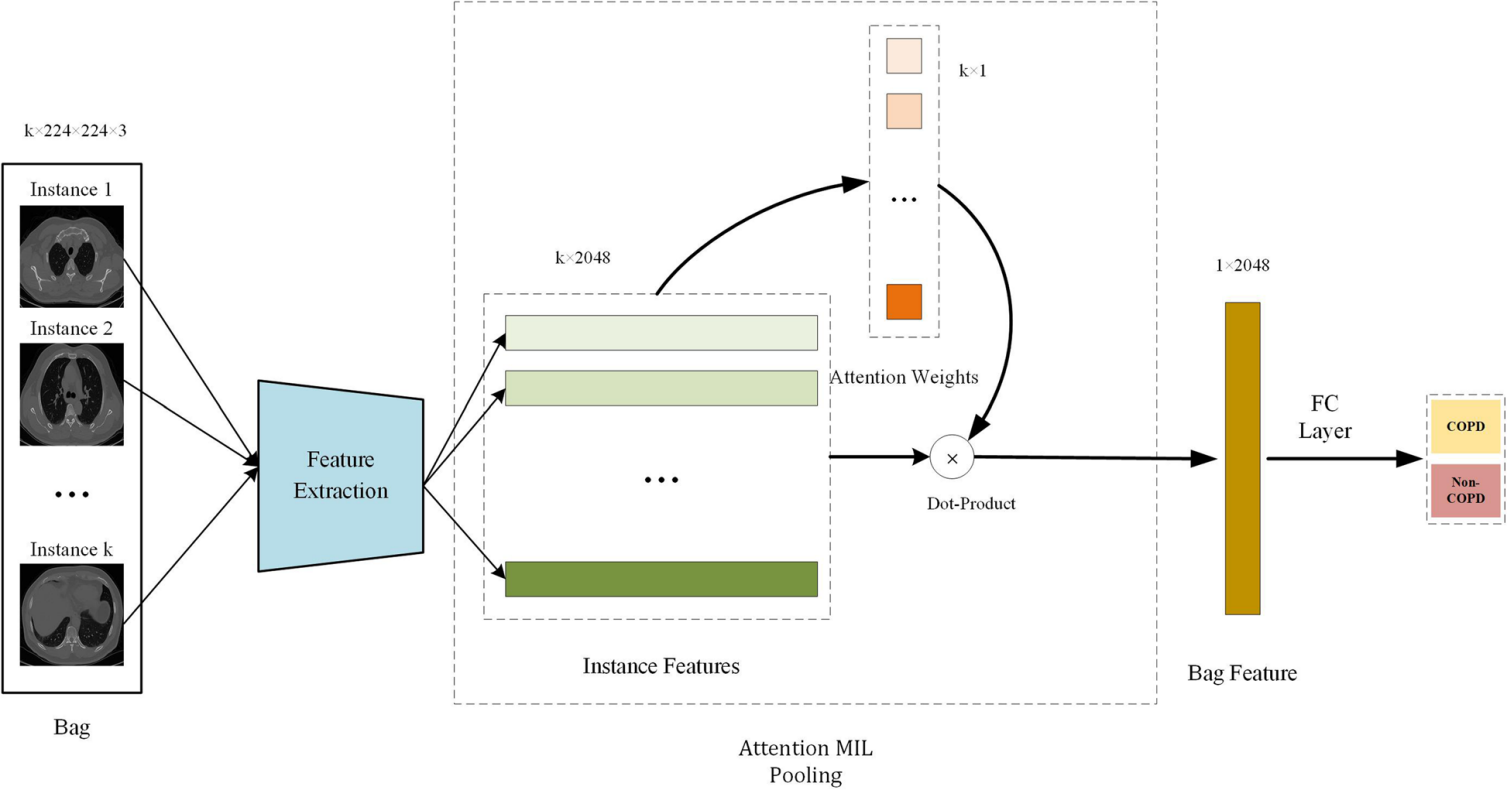
The attention-guided MIL for COPD identification using CT images [32]

Figure 5 depicts the multiple instance earning (MIL) for COPD identification using CT scans. MIL is a weakly supervised learning paradigm where the training data consists of labeled bags, each containing multiple instances [55]. In the context of COPD recognition, a bag corresponds to a CT scan, and instances within the bag represent different regions or patches of the scan. The MIL framework is particularly suitable for COPD recognition because it can handle the heterogeneous nature of the disease, where the presence of COPD may be indicated by local patterns or abnormalities in specific regions of the CT scan. Instead of requiring detailed pixel-level annotations, MIL allows for the learning of COPD patterns from weakly labeled data, where only the overall COPD status of each CT scan is provided.

The MIL-based COPD identification pipeline consists of the following steps: (1) Patch extraction: The CT scans are divided into multiple slices, which serve as instances within the MIL framework. These slices can be extracted using techniques such as sliding windows or unsupervised segmentation methods.(2) Feature extraction: Each patch is transformed into a feature representation using a deep learning model, such as a CNN. The CNN is trained to extract discriminative features that capture the local patterns and abnormalities associated with COPD. (3) Attention-guided instance-level prediction: An attention mechanism is introduced to assign different weights to the instances based on their relevance to the COPD prediction. The attention mechanism learns to focus on the most informative slices while suppressing the less relevant ones. This is achieved by computing attention scores for each instance, which are then used to weigh the instance-level predictions. The attention scores can be learned through a separate attention network or by incorporating attention layers within the MIL framework. (4) Bag-level aggregation: The instance-level predictions are aggregated to obtain a bag-level prediction, representing the overall COPD status of the CT scan. Common aggregation strategies include max pooling, average pooling, or attention-based mechanisms that assign different weights to the instances based on their relevance to the COPD prediction.

The application of MIL for COPD identification offers several advantages. First, it allows for the learning of COPD patterns from weakly labeled data, reducing the need for detailed pixel-level annotations. Second, MIL can handle the heterogeneous nature of COPD, capturing local patterns and abnormalities that may be indicative of the disease. Third, the MIL framework is flexible and can be combined with various deep learning architectures and aggregation strategies to improve the accuracy and robustness of COPD recognition.

## Literature search and review

To compile the necessary literature for this study, two separate reviewers independently scoured Google Scholar, Web of Science, and PubMed for articles applying AI/ML methodologies in COPD-related research, from their inception through November 2023.

A targeted search was conducted on Google Scholar, a well-regarded bibliographic retrieval database, using [“Artificial Intelligence” AND COPD], [“Machine Learning” AND COPD], [“Deep Learning” AND COPD], [“Convolutional Neural Networks” AND COPD], [“Detection” AND COPD], [“Classification” AND COPD], [“Airway” AND COPD], [“Vessel” AND COPD], [“Classification” AND “emphysema”], [“Segmentation” AND “emphysema”], [“Classification” AND “Airway”], [“Segmentation” AND “Airway”], [“Classification” AND “Vessel”], [“Segmentation” AND “Vessel”], [“Segmentation” AND “artery-vein”]. as the main search terms. The same search strings are employed to search on Web of Science and PubMed.

The articles gathered from the three databases were aggregated, and duplicate entries were eliminated. We retained only original research articles written in English. The remaining articles underwent a review process to ensure that only those pertinent to the study were kept.

**Fig. 6:**
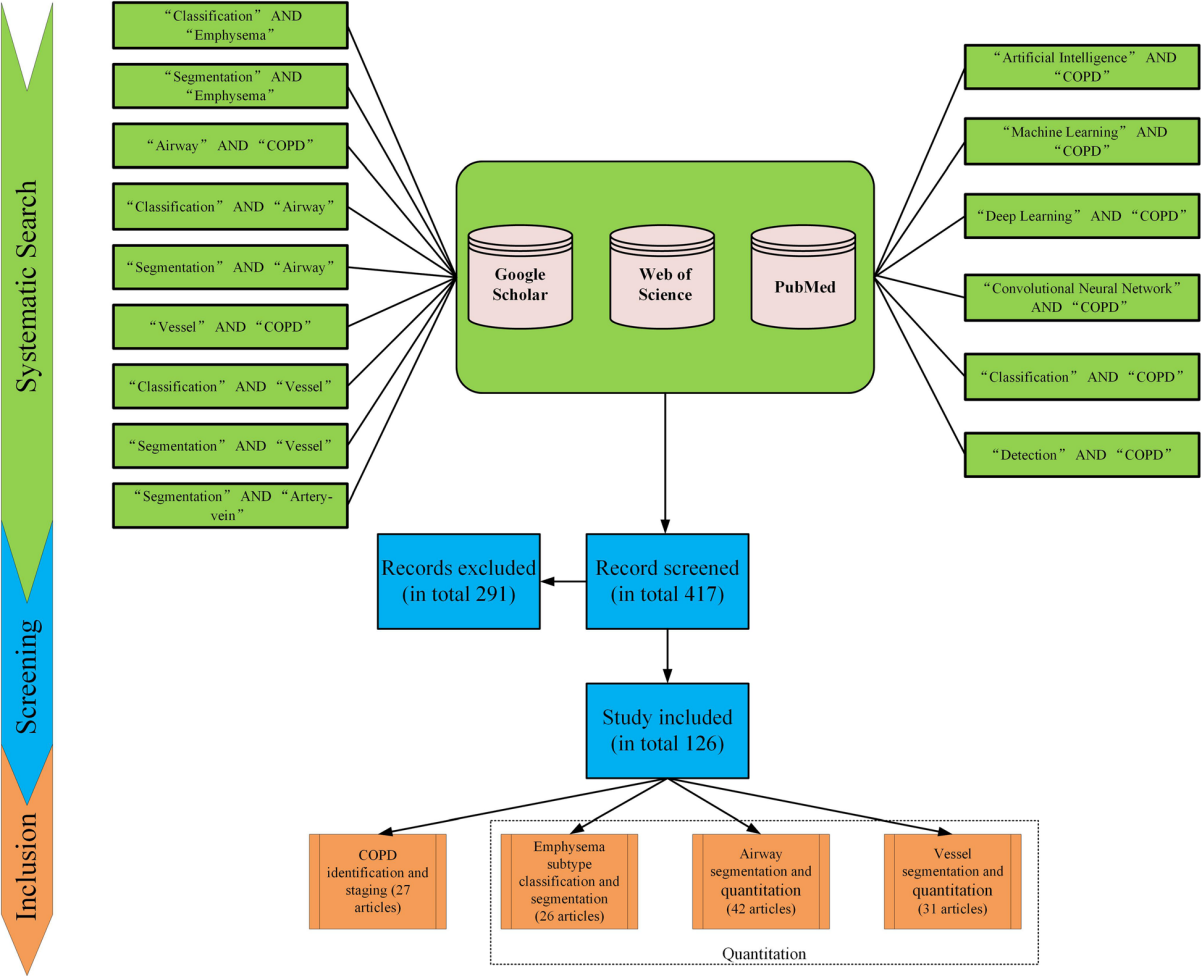
Literature search and analysis. The PRISMA flowchart for this review

Upon finalization, we had a collection of 126 articles. These were classified into four key categories based on their content and objectives: (1) COPD identification and staging (2) emphysema subtype classification and segmentation, (3) airway segmentation and quantification in COPD, and (4) vessel segmentation and quantification in COPD. The step-by-step process is illustrated in Fig. 6.

## AI techniques for COPD identification, staging, and imaging phenotype

Some articles have reviewed the progress of AI techniques in COPD [56–58]. Exarchos et al. reviewed the general adoption of AI in COPD research, categorizing the studies into ‘COPD diagnosis’, ‘COPD prognosis’, ‘Patient classification’, and ‘COPD management’. It identified an acceleration of AI use in COPD research and calls for broader adoption due to the large and complex data involved [57]. The article published by Nikolaou et al. focused on the use of machine learning algorithms, specifically cluster analysis, to better characterize COPD through integration of patient characteristics like symptoms, comorbidities, biomarkers, and genomic information. It reviewed the progress of research in the past decade using cluster analysis for COPD phenotypes [56]. Estépar’s article provided an introduction to AI and deep learning, discussing their role in understanding the evolution and divergent trajectories of COPD. It highlighted the successes of AI in clinical decision making, radiological interpretation, prognostication, and presents opportunities, challenges, and limitations of AI in COPD [58].

Our review article, compared to the others, provides a more in-depth focus on machine learning and deep learning techniques for COPD identification, staging, and imaging phenotypes, emphasizing the roles of emphysema, airway dynamics, and vascular structures. While all articles discuss AI’s role in COPD, we have delved deeper into the specifics of AI-powered insights and the complexities of integrating AI into the clinical landscape. Unlike the other articles, we also provide a comprehensive understanding of the current state and future potential of AI in shaping COPD diagnosis and management.

**Fig. 7:**
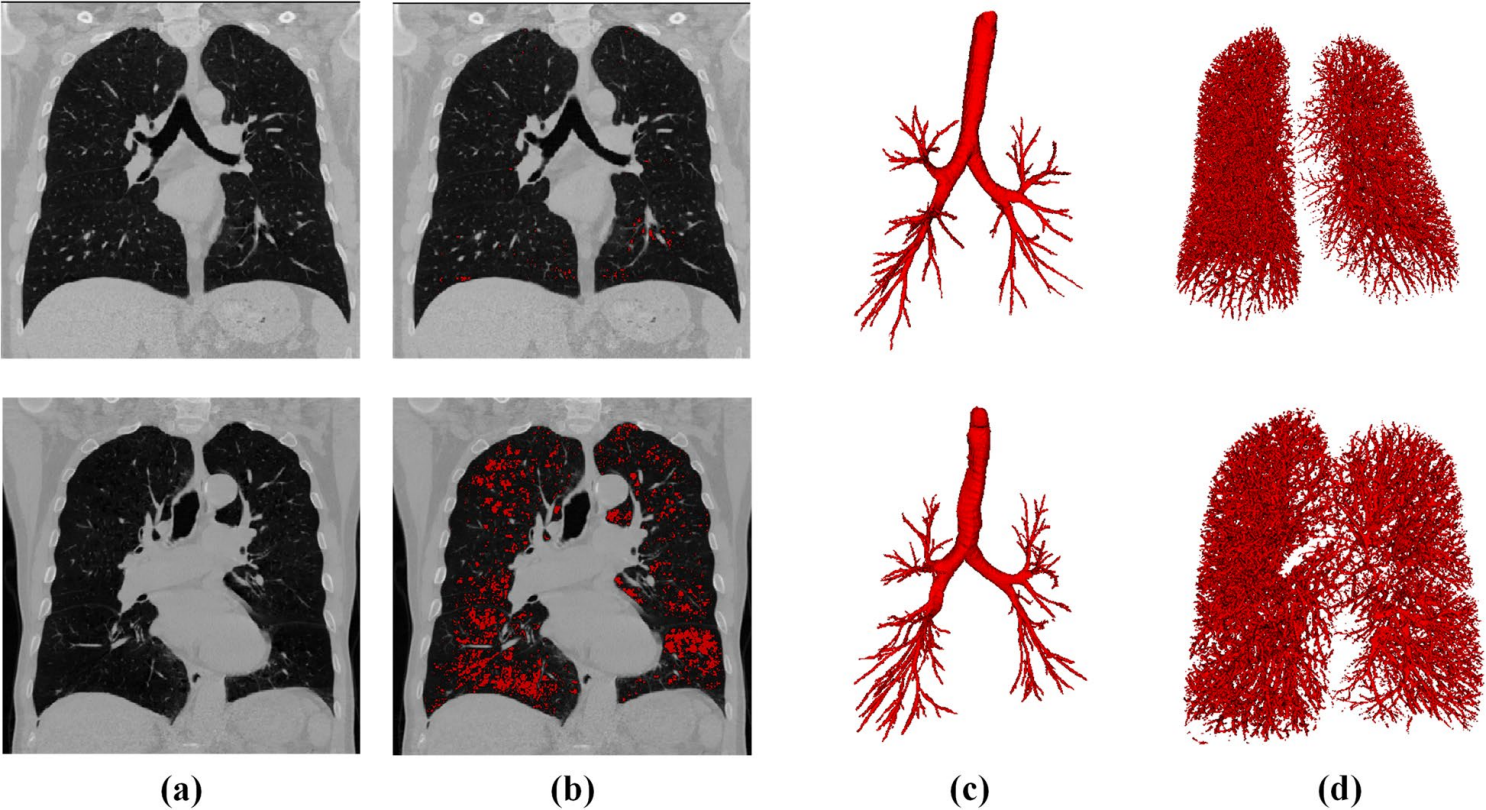
Coronal computed tomography (CT) view, emphysema region(in red), airway and vascular morphology in two COPD subjects with different stages. (Top) Male, Age = 51, BMI = 28.52, GOLD 1, FEV1 = 3.29, FVC = 4.75. (Bottom) Male, Age = 81, BMI = 25.49, GOLD 2, FEV1 = 1.71, FVC = 3.77. Emphysema index was calculated using a threshold of -950 HU in inspiratory CT scan [61]. The lung airway was extracted using the method in [59]. And lung vessel was segmented by [60]. BMI: body mass index; COPD: chronic obstructive pulmonary disease; FEV1: forced expiratory volume in one second; FVC: Forced Vital Capacity; GOLD: Global Initiative for Chronic Obstructive Lung Disease

COPD remains a prevalent respiratory condition, with imaging playing a pivotal role in its diagnosis and management. Figure 7 shows emphysematous destruction, airway, and vascular structure variability in two COPD subjects with different stages obtained with our previous studies [59, 60]. In the following section, we will review the AI in COPD Imaging from four aspects: COPD Identification and Staging, emphysema subtyping, airway analysis, and vascular changes.

### COPD identification and staging with AI

#### Understanding COPD through radiomics: advanced features extracted from CT images

All of the papers related to COPD identification and staging with AI are summarized in Table 1. Radiomics, an emerging field in medical imaging, involves the extraction of a large number of quantitative features from radiographic images [62]. In the context of COPD, radiomics can provide numerous features from CT scans, capturing detailed information about lung morphology and texture that can indicate disease presence and severity [63]. For example, first order features describe the distribution of voxel intensities within the image through basic metrics such as mean, variance, skewness, and kurtosis. Shape features provide information about the 3D shape of the lung and its irregularities. Matrix-based features like the gray level co-occurrence matrix (GLCM), gray level size zone matrix (GLSZM), gray level run length matrix (GLRLM), neighboring gray tone difference matrix (NGTDM), and gray level dependence matrix (GLDM) describe more complex characteristics. The GLCM quantifies the texture by examining the spatial distribution of voxels. GLSZM captures the size zones of different gray levels, while GLRLM gives information about the lengths of consecutive voxels with the same gray level. NGTDM measures the difference between a voxel and its neighbor, and GLDM quantifies the dependencies of gray level values in the image.

**Table 1:** Summary of COPD identification and staging by different teams

Year	Reference	Team	Dataset	Task	Main methods	Performance
2013	[64]	Mets et al.	1140 inspiratory and expiratory CT	COPD identification	- The segmentation of lung and airway	ACC=82.8%, SEN=88%, SPE=73.2%, PPV=80.2%, NPV=84.2%
			437 COPD		- Three quantitative CT biomarkers (emphysema, air trapping, and bronchial wall thickness)	
					- Logistic regression	
2016	[65]	Ying et al.	COPDGene	COPD GOLD classification (GOLD 2011)	- Fisher score method	ACC=97.2%
					- Deep belief network	
2017	[66]	González et al.	COPDGene: 3881 COPD vs 4387 non-COPD, ECLIPSE: 1727 COPD	COPD identification and stage	- Four canonical views of the CT scan	Identification:
					- CNN with three convolutional layers	(COPDGene) ACC=77.3% AUC= 0.856
						(ECLIPSE) AUC=0.548
						Stage:
						(COPDGene) ACC=51.1%
						(ECLIPSE) ACC=29.4%
2017	[67]	Cheplygina et al.	-Danish Lung Cancer Screening (DLCST)	COPD identification	- Gaussian texture features	DLCST: AUC=0.684
			-Partial COPDGene		- Multiple instance learning	COPDGene: AUC=0.962
					- Transfer learning	Frederikshavn AUC=0.969
2018	[68]	Sathiya et al.	—	COPD identification	- Gray Level Co-occurrence Matrix	—
					- Fuzzy c-means clustering	
					- CNN classifier	
2020	[69]	Singla et al.	COPDGene	GOLD diagnosis and stage	- Discriminative network	Diagnosis: AUC=0.82 Recall=0.80
					- Attention mechanism	Stage: ACC=65.44%
					- Generative network	
2020	[70]	Xu et al.	190 COPD vs 90 non-COPD	COPD identification	- CT lung image	ACC=99.29%, AUC=0.9826, SEN=99.47%
					- The fourth convolutional layer for 9 feature extraction Principle component analysis MIL, Citation-KNN for classification	SPE=98.89% F1-score=0.9947
2020	[71]	Du et al.	190 COPD vs 90 non-COPD	COPD identification	- Snapshots of different view of airway tree	ACC=88.6%
					- CNN with Bayesian optimization	
					- Majority voting	
2020	[72]	Tang et al.	1304 COPD vs 1285 non-COPD (PanCAN)	COPD identification	- CT images	PanCAN: AUC=0.889
					- ResNet 152	ECLIPSE: AUC=0.886
						PPV=0.847, NPV=0.755
2021	[73]	Ho et al.	204 COPD vs 392 non-COPD	COPD identification	- Parametric-response mapping of CT image	ACC=89.3%, SEN=88.3%, AUC=0.937
					- 3D CNN	
2021	[74]	Hasenstab et al.	COPDGene.	GOLD stage	- Co-registration	SEN=88.25%, SPE=74.5%, AUC=0.905
					- Lung segmentation	
					- Measurements of emphysema and air trapping	
					- Logistic regression	
2022	[75]	Chen et al.	707 COPD vs 4116 non-COPD	COPD identification	- Graph convolutional MIL	AUC=0.96
					- Adaptive additive margin loss	
2022	[76]	Li et al	249 patients with stable COPD and 73 controls	COPD identification and stage	- Radiomics	Identification:
					- Feature selection methods, including variance threshold, Select K Best method, and least absolute shrinkage and selection operator (LASSO)	ACC=0.941, SEN=0.940
					- Support vector machine (SVM)	SPE=0.955, AUC=0.970
						Stage:
						ACC=0.759, SEN=0.723
						SPE=0.805, AUC=0.799
2022	[77]	Makimoto et al.	602 COPD, 602 non-COPD	COPD identification	- Resampling, segmenting the lung and removing the airways	AUC=0.78
					- Radiomics	
					- Elastic Net (feature selection)	
					- SVM	
2022	[78]	Zhang et al.	204 COPD vs 392 non-COPD	COPD identification	- Lung parenchyma and bronchial wall patch of CT lung images	ACC=81.7%, SEN=81.0%
					- DenseNet-201	SPE=81.6%, AUC=0.899
2022	[37]	Wu et al.	291 COPD vs 290 non-COPD	COPD identification	- Snapshots of different view of airway tree and lung field	ACC=94.7%
					- ResNet-26	SEN=92.9%
					- Majority voting	SPE=96.7%
2022	[38]	Sun et al.	749 non-COPD vs 644 COPD	COPD identification and stage	- CT images	Identification
					- ResNet18, attention MIL	SEN=80.5%, SPE=92.5%, AUC=0.934
					- Multi-channel 3D residual network	Stage:
						SEN=76.5%, SPE=92.2%, AUC=0.912
2022	[79]	Li et al.	204 COPD vs 392 non-COPD	COPD identification	- Segmented lung parenchyma of CT images	ACC=77%, Precision=0.80
					- Graph convolutional network	F1-score=0.78, AUC=0.81
2022	[80]	Yang et al.	468 subjects with Stage 0 to IV	COPD identification and stage	- Radiomics	ACC=0.80, Precision=0.943
					- LASSO	F1-score=0.946, AUC=0.94
					- Multi-layer perceptron	
2022	[81]	Yang et al.	465 subjects with Stage 0 to IV (129, 108, 121, and 107)	COPD identification and stage	- Radiomics, 3D CNN features	ACC=0.943, Precision=0.943
					- Auto-metric graph neural network	F1-score=0.946, AUC=0.984
2023	[32]	Wu et al.	271 COPD vs 290 non-COPD	COPD identification	- CT images, Snapshots of different view of airway tree and lung field	ACC=95.8%
					- Attention MIL, LR	SEN=95.3%
						SPE=96.5%
2023	[82]	Almeida et al.	COPDGene and COSYCONET [83]	COPD identification	- Spatial alignment, Lung. Traches, and aorta segmentation (Pre-processing)	COPDGene:
					- Self-supervised learning	AUC=0.843
					- Generative Model	COSYCONET:
						AUC=0.679
2023	[84]	Xue et al.	363 COPD vs 437 non-COPD	COPD identification	- Transfer learning (pre-trained Resnet-50)	ACC=92%, SEN=92%
					- Pseudo-color method	SPE=91.95%, AUC=0.9544
					- Two stage attention MIL	
2023	[85]	Zhou et al.	COPD (4,912),	COPD identification	- Multi-modal (Radiograph, chief complaint, and demographics and lab test results)	AUC=0.922
					- Transformer-based representation and classification	
2023	[86]	Puchakayala et al.	COPDGene	COPD identification	- Demographics features, emphysema and radiomics features of CT images	Standard-Dose CT Data:
					- CatBoost	PPV=0.86, NPV=0.83, AUC=0.90
						Low-Dose CT Data:
						PPV=0.79, NPV=0.80, AUC=0.88
2023	[87]	Yu et al.	COPDGene	COPD stage	- Self-supervised Learning	ACC=0.65
					- CNN	

Cheplygina and his colleagues demonstrated that machine learning methods could effectively utilize radiomics features to classify and detect COPD in chest CT images [67]. The proposed method had validated on multi-dataset and achieved a moderate area under the curve (AUC) (DLCST:0.684, COPDGene: 0.962, and Frederikshavn: 0.969). Li et al. showed that lung radiomics features, when combined with a support vector machine (SVM) model, could accurately classify different stages of COPD, outperforming conventional methods, achieving 0.970 of AUC in identification and 0.799 AUC in staging [76]. According to the study, machine learning methods, coupled with radiomics features, accurately classified the stages of COPD and outperformed classical CNN [77]. Yang et al. explored how a multi-layer perceptron classifier, coupled with selected lung radiomics features, can effectively characterize and classify COPD stages [80]. Moreover, he proposed a novel lung radiomics combination vector and an auto-metric graph neural network with a meta-learning strategy for effective COPD stage classification [81]. Amudala’s study suggested that a combination of radiomics features, representing parenchymal texture and lung and airway shape of CT images, could be used to accurately detect COPD [86]. The proposed method gain tremendous classification potential in inspiratory low-dose (AUC=0.88) and standard-dose CT scans (AUC=0.90) .

#### Deep learning and COPD: unveiling disease markers from CT images

Deep learning techniques, especially CNNs, have shown remarkable success in image analysis tasks. CNNs have the capability of automatically learning hierarchical features from raw CT images, which can capture both primitive (edges, textures) and more abstract features (shapes, structures) in the data. In the context of COPD, CNNs can be trained to identify structural changes in the lung indicative of the disease, including emphysema, airway thickening, and the presence of mucus plugs. They can also be used to quantify the extent of these changes, providing a measure of disease severity. The advantage of deep learning lies in its ability to learn from large-scale data, capturing subtle changes and complex patterns that might be overlooked by human experts or traditional image processing techniques.

In COPD identification, Sathiya et al. presented a computer-aided diagnosis system for COPD that employed CNN to classify CT images, with a process encompassing preprocessing, feature extraction, segmentation, and classification, aiming to enhance the accuracy of COPD diagnosis under noisy conditions [68]. Tang and his colleagues demonstrated the effectiveness of deep residual networks for the automated detection of COPD using low-dose CT scans, achieving an AUC of 0.889 in PanCAN and 0.886 in ECLIPSE [72]. Li et al. presented a novel method for early COPD detection using a graph convolution network model applied on small and weakly labeled chest CT images [79]. It gained robust classification performance (AUC:0.81). Moreover, deep CNN transferred Multiple Instance Learning (DCT-MIL) approach [32, 70, 84] and self-supervised learning [82, 87] were employed to identify COPD from CT images. In COPD staging, Ying et al. utilized a deep belief network to develop a highly accurate automatic classifier for COPD severity, demonstrating its effectiveness as a tool for exacerbation risk assessment in COPD patients (accuracy (ACC)=97.2%) [65]. While another study developed a deep learning-based algorithm to stage COPD severity through quantification of emphysema and air trapping from CT images, showing that automated CT algorithms can facilitate COPD severity staging and provided prognostic value [74]. The proposed method achieved remarkable discriminative capacity in COPD staging with a sensitivity (SEN) of 88.25%, a specificity (SPE) of 74.5%, and an AUC of 0.905.

Some works propose deep learning methods for both identification and staging. Gonzalez et al. demonstrated that the several layers’ CNN could effectively detect and stage COPD, predict acute respiratory disease events and mortality in smokers using only CT imaging data, suggesting that CNN analysis could be a powerful tool for risk assessment at a population level [66]. Singla and his colleagues developed a deep learning model to extract informative regional image features from HRCT of COPD patients, demonstrating strong predictive capabilities for spirometric obstruction, emphysema severity, exacerbation risk, and mortality, and potentially improving both research and clinical practice [69]. Moreover, the CNN models which were trained using images of lung parenchyma and bronchial wall [78] and weakly supervised deep learning [38] to diagnose and grade COPD.

In the deep learning methods for COPD identification and staging, researchers have employed various neural network architectures, numbers of layers, and activation functions. Based on the information provided in Table 2, the following three points can be summarized: CNNs are the most commonly used neural network architecture in COPD identification and staging. Researchers have utilized various classic CNN architectures, such as AlexNet, ResNet, DenseNet, and VGG, as well as some self-designed CNN architectures. These architectures typically consist of multiple convolutional layers and pooling layers to extract features from the images. The number of layers in the neural networks varies considerably, ranging from as few as 3 layers to as many as 201 layers. Some studies have used shallower networks, such as González et al. [66] and Ho et al. [73], who employed CNNs with 3 convolutional and pooling layers. In contrast, other studies have utilized deeper networks, such as Zhang et al. [78] with DenseNet-201 (201 layers) and Tang et al. [72] with ResNet-152 (152 layers). The Rectified Linear Unit (ReLU) is the most frequently used activation function in these deep learning models for COPD identification and staging. Most of the studies listed in the table have employed ReLU as their activation function, with the exception of Du et al. [71] who used Leaky ReLU and Yu et al. [87] who used Sigmoid. The choice of activation function can impact the model’s ability to learn complex patterns and generate meaningful representations of the input data.

**Table 2:** More details of deep learning method in COPD identification and stage, including neural network architectures, number of layers, and activation functions

Team	Reference	Neural network architecture	Number of layers,	Activation functions
González et al.	[66]	CNN	Three conv and max pooling	Rectified Linear (ReLU)
Xu et al.	[70]	AlexNet	Five conv and three max pooling	ReLU
Du et al.	[71]	Self-designed	Some conv and max pooling	Leaky ReLU
Tang et al.	[72]	ResNet 152	152	-
Ho et al.	[73]	3D CNN-Naive	Three conv and max pooling	ReLU
Zhang et al.	[78]	DenseNet-201	201	ReLU
Wu et al.	[37]	ResNet-26	26	ReLU
Sun et al.	[38]	ResNet-18	18	ReLU
Wu et al.	[32]	VGG-16	16	ReLU
Almeida et al.	[82]	3D ResNet-34	34	-
Xue et al.	[84]	Resnet-50	50	ReLU
Yu et al.	[87]	Loc-CondConv	-	Sigmoid

In the preprocessing, feature extraction, and feature selection methods employed in deep learning approaches for COPD identification and staging, researchers have utilized various techniques to enhance the performance of their models. Based on the information provided in Table 3, the following three points can be summarized: (1) Preprocessing techniques, such as segmentation of the lung and airways, are commonly used to focus the analysis on the relevant regions of interest in the CT images. Several studies, including Mets et al. [64], Xu et al. [70], Hasenstab et al. [74], and Puchakayala et al. [86], have employed lung and airway segmentation as a preprocessing step. Other preprocessing methods include joining multiple views into a single montage (González et al. [66]), extracting 3D regions of interest (Cheplygina et al. [67]), and applying grayscale conversion (Sathiya et al. [68]).(2) Feature extraction methods can be broadly categorized into two main approaches: (a) using pre-trained or self-designed CNN architectures to automatically learn relevant features from the preprocessed images, and (b) calculating handcrafted features, such as radiomics features or specific quantitative CT biomarkers. Studies by González et al. [66], Xu et al. [70], and Tang et al. [72] have utilized CNN-based feature extraction, while others, such as Mets et al. [64], Hasenstab et al. [74], Li et al. [76], Yang et al. [80, 81], and Puchakayala et al. [86], have employed handcrafted features. (3) Feature selection methods are occasionally used to reduce the dimensionality of the extracted features and select the most informative ones for COPD identification and staging. Some studies, such as Xu et al. [70], have used principal component analysis (PCA) for feature selection, while others, like Li et al. [76] and Yang et al. [80, 81], have employed techniques such as variance threshold, Select K Best method, least absolute shrinkage and selection operator (LASSO), and generalized linear models. However, not all studies have explicitly mentioned the use of feature selection methods, suggesting that the choice of feature selection techniques may depend on the specific requirements and characteristics of the dataset and the model being used.

**Table 3:** The preprocessing, feature extraction, and feature selection method of deep learning method in COPD identification and stage

Team	Reference	Preprocessing	Feature extraction	Feature selection
Mets et al.	[64]	The segmentation of lung and airway	Three quantitative CT biomarkers (emphysema, air trapping, and bronchial wall thickness)	-
González et al.	[66]	Join four views into a single montage	CNN features	-
Cheplygina et al.	[67]	3D Region of interest (ROI) from CT image	Gaussian scale space features	-
Sathiya et al.	[68]	Gray Scale	Gray Level Co-occurrence Matrix	-
Xu et al.	[70]	The segmentation of lung from CT image	CNN features (AlexNet)	Principle component analysis
Tang et al.	[72]	Lung mask generation, spatial normalisation	CNN features (ResNet-152)	-
Hasenstab et al.	[74]	Co-registration, lung segmentation	Emphysema and air trapping feature	-
Li et al.	[76]	Volume of Interest segmentation from CT	1395 radiomics features	Variance threshold, Select K Best method, and least absolute shrinkage and selection operator (LASSO)
Yang et al.	[80]	Lung region segmentation	1316 radiomics features	LASSO
Yang et al.	[81]	Lung parenchyma segmentation	1316 radiomics features	Generalized linear model and LASSO
Puchakayala et al.	[86]	Segmentation of lung and airways	Demographics features, emphysema feature, lung and airway radiomics features	-

#### Beyond traditional methods: unique feature extraction from CT images for COPD identification and staging

Other innovative features can be extracted from CT images for COPD identification . For instance, the extraction of the bronchial tree structure provides a unique, detailed representation of the airways. Analyzing the branching patterns, diameters, and wall thicknesses of the bronchial tree can reveal valuable insights into the disease [78]. Additionally, A novel method diagnosed COPD using deep CNN to assess snapshots of 3D airway trees extracted from CT images [71] with an accuracy of 88.6%. Moreover, Wu and his colleagues further utilized snapshots of 3D airway trees and lung fields for COPD identification [37], achieving a higher accuracy of 94.7%. By combining these diverse features and original CT image features, They also created a comprehensive representation of the lung that can potentially improve the performance of machine learning models in COPD identification [32] with the noteworthy classification performance (ACC:95.8%, SEN:95.3%, and SPE:96.5%). Other study developed a new classification method for COPD based on CNN and a parametric-response mapping with an accuracy of 89.3% [73]. Future research should focus on developing effective feature integration strategies and exploring the potential of novel imaging features.

### Emphysema imaging analysis through AI lenses

Emphysema is characterized by the irreversible destruction of alveoli, the lung’s air sacs. CT imaging is instrumental in its detection and quantification. However, with the integration of AI, the landscape of emphysema identification, subtyping, and phenotypic visualization is undergoing transformative changes [88].

#### Characteristics and subtypes

**Fig. 8:**
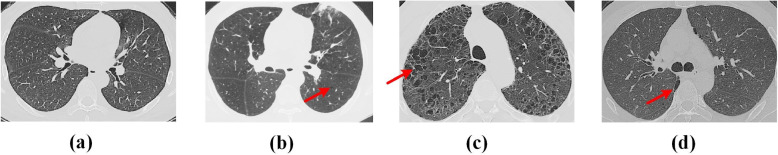
Examples of different lung tissue patterns are indicated by the red arrow.(a)Normal lung parenchyma (NLP);(b) centrilobular emphysema (CLE);(c) panlobular emphysema (PLE); and (d) paraseptal emphysema (PSE)

In emphysema, alveoli are damaged and enlarged causing breathlessness. CT scans, particularly high-resolution CT (HRCT), play a crucial role in diagnosing and characterizing emphysema. As defined in [10], emphysema can be categorized into several subtypes based on the distribution and appearance of the disease on CT scans (as shown in Fig. 8): **centrilobular emphysema (CLE)** is the most common type of emphysema seen in smokers. It typically begins in the center of the secondary pulmonary lobule, primarily affecting the bronchioles while sparing the peripheral portion. On CT images, it appears as areas of low attenuation without visible walls, often in an upper lobe predominant distribution. **Panlobular emphysema (PLE)** involves the entire secondary lobule. It is most commonly associated with $\alpha$1-antitrypsin deficiency. On CT scans, it presents as diffuse, low attenuation areas affecting all lung zones, but more severe in the lower lobes and anterior lung zones. **Paraseptal emphysema (PSE)** is characterized by the involvement of the distal airway structures adjacent to the pleura. It is often found in the upper lobes. On CT, it appears as subpleural areas of low attenuation, often with visible walls, and may be associated with bulla formation. In all these types, the degree of emphysema can be quantified on CT through densitometric analysis, which measures the lung’s mean attenuation and the percentage of the lung volume with low attenuation. These quantitative measurements can help assess the severity of the disease and monitor its progression. Moreover, the severity of emphysema is often categorized based on the percentage of the lung volume that falls below a certain Hounsfield Unit (HU) threshold on CT. It’s important to note these are general guidelines and the specific thresholds can vary depending on the source [89].

#### AI-based severity identification and subtype classification

AI has enhanced the diagnostic horizon for emphysema in multiple ways (Table 4):

**Table 4:** Summary of emphysema subtype classification methods by different teams, NT:normal tissue, CLE: centrilobular emphysema, PLE: panlobular emphysema, and PSE: paraseptal emphysema

Team	Reference	Year	Task	Method Keypoints	Metrics
Gangeh et al.	[90]	2010	NT, CLE, and PSE	- Texton-based features (k-means)	ACC=96.43%, SEN=95.41%, SPE=98.31%
				- KNN and SVM	
Sørensen et al.	[91]	2010	NT, CLE, and PSE	- Local binary pattern	ACC=95.2%
				- KNN	
Zulueta-Coarasa et al.	[92]	2013	NT, CLE1, CLE2, CLE3, PLE, and PSE	- Embedded probabilistic PCA	Precision=0.72, SEN=0.73, SPE=0.95
				- Maximum a Posterior	
Karabulut et al.	[93]	2015	NT, CLE, and PSE	- Patches from HRCT	ACC=84.25%
				- CNN	
Peng et al.	[94]	2017	NT, CLE, PLE, and PSE	- Joint Weber-based rotation invariant LBP	ACC=95.83%
				- KNN	
Bortsova et al.	[95]	2018	CLE, PSE	- Lung region of CT image	SEN=0.65, SPE=0.95, AUC=0.89
				- MIL	
Bermejo-Peláez et al.	[96]	2018	NT, CLE1, CLE2, CLE3, PLE, and PSE	- 2.5D CNN (4 convolutional, 3 max-pooling layers and 3 fully-connected layers)	SEN=81.78%, SPE=97.34%, AUC=97.25%
Bermejo-Peláez et al.	[97]	2019	PSE	- Volume of 384 $\times$ 384 $\times$ 8 voxels in CT image	Dice similarity coefficient (DSC)=0.764
				- Slice-Recovery network (3D CNN)	
Peng et al.	[98]	2019	NT, CLE, PLE, and PSE	- 2D patch of CT images	ACC=93.74%
				- Multi-scale CNN (20-layer ResNet)	
Peng et al	[99]	2019	NT, CLE, PLE, and PSE	- Semi-supervised learning	ACC=82.6%
				- CNN	
				- Fisher Loss	
Wu et al.	[52]	2021	NT, CLE, PLE, and PSE	- 2D patch of CT images	ACC=95.95%,SPE=98.85%, AUC=0.99
				- Vision Transformer	Precision=96.38%, Recall=96.58%
Li et al.	[100]	2021	NT, CLE, and PSE	- Local quinary pattern, fractal features and intensity histograms	ACC=92.3%
				- Autoencoder, PCA (feature selection)	
				- SVM (classifier)	
Ørting et al.	[101]	2018	Emphysema detection	- MIL	AUC=0.82
Humphries et al.	[102]	2019	Emphysema severity	- CNN-LSTM	-
Mondal et al.	[103]	2021	Emphysema severity	- Weber Local Binary Pattern	ACC=93.75%
				- CNN	

**Enhanced sensitivity with machine learning and traditional features**: Machine learning models, trained on texton features, are proficient in identifying subtypes of emphysema. Gangeh et al. introduced a new texton-based classification system, coupled with a SVM model, for classifying emphysema in CT lung images, demonstrating superior accuracy (96.43%) over common techniques and slight improvement over recent methods based on local binary patterns [90]. Other methods including local binary patterns and joint intensity histograms [91], ensemble features based on log-Gabor filters, mean difference technique, and intensity values [104], representation by rotation invariant uniform local ternary pattern with Weber’s law [94], combination of local quinary patterns, multifractal features, and intensity histograms [100], was proposed for emphysema subtyping. Moreover, Zulueta et al. explored the use of a manifold learning technique with embedded probabilistic PCA for classifying different types of emphysema in CT lung images, demonstrating competitive performance with traditional texture-based and intensity distribution methods, as well as good visual agreement with actual emphysema types in full lung analysis [92].

**Automatically extract features with deep learning**: CNNs can identify and isolate emphysematous regions without the need for manual feature engineering, thus capturing the intricate patterns indicative of the disease. Karabulut et al. explored a CNN model to automatically identify and discriminate between subtypes of emphysema in high-resolution CT lung images, demonstrating promising accuracy levels and reduced processing time [93]. Peng and his colleagues presented a novel multi-scale residual network for automated emphysema tissue classification, achieving 93.74% accuracy, and introduced a new measure of emphysema severity based on the sum of centrilobular and panlobular emphysema, demonstrating strong correlation with pulmonary functions [98]. As illustrated in the work by Bermejo’s group, the Slice-Recovery network (SR-Net) was introduced, which was a novel convolutional network architecture that utilizes 3D contextual information for 2D segmentation of PSE lesions in CT images with the dice of 0.764 [97]. In the study led by Wu, they proposed a vision Transformer (ViT) [54] model for the classification of emphysema subtypes based on CT images, using large patches cropped from the images for embedding and classification. Using pre-training on ImageNet to overcome data limitations, the ViT model achieved an average accuracy of 95.95% on a proprietary dataset, outperforming AlexNet [33], Inception-V3 [105], MobileNet-V2 [106], ResNet34 [30], and ResNet50, as well as a non-pretrained ViT model. These results suggested that the proposed ViT model can accurately classify emphysema subtypes and had potential for other medical applications [52].

**Automated evaluation the severity of emphysema**: AI algorithms can categorize the severity of emphysema by automatically quantifying the affected lung volume, providing a more objective and consistent staging compared to manual densitometry. Bortsova et al. introduced an end-to-end deep learning method to estimate the extent of emphysema based on the proportion of diseased tissue. It outperforms traditional lung densitometry and other recent methods by a significant margin with an AUC of 0.89 [96].

**Segmentation using deep learning**: Deep learning, especially CNNs, also has demonstrated a marked aptitude for emphysema segmentation in CT scans. As reported by Peng and his fellow researchers introduced a new end-to-end semi-supervised framework for the semantic segmentation of emphysema in CT images using both annotated and unannotated areas. It was designed to reduce the workload for radiologists and annotation workload. The authors also propose a new loss function, the Fisher loss, to improve the model’s discriminative power. Experimental results demonstrate that this approach outperforms both the baseline supervised approach (which uses only annotated areas) and other state-of-the-art methods for emphysema segmentation ACC=82.6% [99].

**Derived subtypes**: Yang and his team investigated the possibility of using texture learning to identify novel emphysema specific lung texture patterns (sLTPs), which might correspond to previously unrecognized emphysema subtypes with distinct clinical traits. They employed advanced clustering techniques on emphysematous region textons within the MESA COPD cohort 49, pinpointing 12 unique sLTPs. A notable feature of this method was its incorporation of spatial data, since the regional distribution of emphysema is considered a significant phenotype 50. While most sLTPs displayed a strong correlation with dyspnea and exercise capacity, further research is essential to fully grasp their pathological significance [107, 108].

In conclusion, the incorporation of AI, particularly deep learning, has redefined the paradigms of emphysema detection and analysis. These advancements underscore the immense potential of AI in tailoring therapeutic interventions and prognostic assessments for patients with emphysema.

### Airway analysis with AI

The airways, comprising bronchi and bronchioles, undergo significant structural changes in the face of COPD. HRCT imaging captures these alterations. AI’s integration with radiology accentuates the precision, scale, and depth of airway analysis, amplifying our understanding and management of COPD.

#### From visual cues to machine insights

Traditionally, radiologists discern airway changes by identifying bronchial wall thickening, luminal narrowing, and mucous plugging in scans [109]. While these visual cues remain foundational, they often necessitate expert scrutiny and can be subjective. AI bridges this gap by two aspects: **Enhancing detection**: AI models, trained on diverse datasets, unearth subtle airway alterations, magnifying early detection prospects [110, 111]. **Standardizing evaluations**: Algorithms ensure consistent airway analysis, minimizing inter-observer variability that can arise from manual evaluations [112, 113].

#### Segmentation and quantification: using AI to segment and measure airway changes

Segmenting the intricate airway structure and quantifying its changes is a complex endeavor. AI shines in this domain by precise segmentation. As shown in Table 5, deep learning algorithms, including CNNs and Transformer, can meticulously delineate airway structures from adjacent lung parenchyma, ensuring accuracy [114–120]. Charbonnier et al. improved the airway segmentation quality by detecting and removing leaks using a convolutional network, and combining multiple segmentations to increase the airway tree length (65.4%) in EXACT’09 while minimizing leaks [121]. A novel 2.5D convolutional neural network-based method was proposed for airway segmentation in volumetric chest CT scans with the tree length of 60.1% and a false positive rate (FPR) of 4.56% [122, 123]. Another research introduced AirwayNet, an innovative voxel-connectivity aware approach for precise airway segmentation in CT scans with a DSC of 90.2%. By transforming the conventional binary segmentation task into 26 connectivity prediction tasks, AirwayNet learns not only the airway structure but also the relationship between neighboring voxels [124]. Nadeem and his colleagues presented a novel multi-parametric freeze-and-grow propagation approach for automated and accurate segmentation of pulmonary airway trees in CT scans for exploring COPD sub-phenotypes. A CT intensity-based FG algorithm and a deep learning-based version are developed [125]. Graph neural network was also employed for extracting airways from chest CT data [126, 127]. A coarse-to-fine framework was proposed for addressing challenges in small airway branch segmentation [59, 128, 129]. Moreover, Wu et al. employed a novel 3D contextual transformer for accurate airway segmentation, extracting significantly more branches and longer lengths of the airway tree [59] with the tree length of 79.6% and a FPR of 8.27% on EXACT’09 dataset . Also, multi-task segmentation including airway and vessel was finished using CNNs-based methods [129–131]. Moreover, topology-guided iterative self-learning approach [132] and long-term slice propagation method [133] were proposed for improving tree length and branch detection.

**Table 5:** Summary of airway segmentation methods by different teams

Team	Reference	Year	Method Keypoints	Datasets	Metrics
Charbonnier et al.	[121]	2016	- Leak detection	45 scans from COPDGene, EXACT’09	COPDGene: ACC=0.97, SPE=0.97, SEN= 0.9
			- CNN		EXACT’09: tree length=65.4% FPR=1.68%
Yun et al.	[122]	2018	- Three adjacent slices in axial, sagittal, and coronal view	Korean obstructive lung disease (KOLD) cohort, EXACT’09	KOLD: tree length=92.16% False Positive Rate (FPR)=7.74%, DSC=0.8997
			- 2.5D CNN		EXACT’09: tree length=60.1% FPR=4.56%
Qin et al.	[124]	2019	- Voxel-connectivity aware CNN	30 CT scans	DSC=90.2%, TPR=84.7%, FPR=0.008
Nadeem et al.	[125]	2020	- Freeze-and-Grow Algorithm CNN	SPIROMICS (COPD study)	-
Selvan et al.	[126]	2020	- Mean-field approximation	Danish lung cancer screening trial	tree length=81.9% FPR=7.8%, Dice=84.8%
			- GNN		
Zheng et al.	[116]	2021	- Group supervision	EXACT’09, Binary Airway Segmentation Dataset (BAS)	EXACT’09: branch count=80.5%, tree length=79.0%, precision=94.2%
			- CNN		BAS: branch count=88.7%, tree length=92.5%, precision=91.4%
			- General Union loss		
Guo et al.	[128]	2021	- Atrous spatial pyramid pooling	Private dataset, EXACT’09	Private dataset: DSC=93.5%, Intersection over Union (IoU)=87.8%, FPR=0.015%, SEN=90.8%
			- CNN-based region growing		EXACT’09: DSC=95.8%, IoU=91.9%, FPR=0.053%, SEN=96.6%
Qin et al.	[130]	2021	- Feature recalibration	EXACT’09, BAS	EXACT’09: branch count=82.0%, tree length=79.4%, FPR=9.71%
			- Attention distillation		BAS: Branch Detected (BD)=82.0%, True Detected (TD)=79.4%,
			- CNN		True Positive Rate (TPR)=93.6%, FPR=0.035%, DSC=92.5%
Heitz et al.	[129]	2021	- Axial, coronal and sagittal slices of CT image	Private dataset	Dice=78.5%
			- 2.5D U-Net		
Cheng et al.	[115]	2021	- Tiny atrous convolutional network (3D CNN)	Private dataset, EXACT’09	Private dataset: Dice=0.9032, BD=86.63%, FPR=1.44%
					EXACT’09: BD=84.9%, TD=84.5%, FPR=14.29%
Huang et al.	[120]	2022	- Adaptive hard region-aware net	EXACT’09, LIDC	Dice=0.912
			- Voxel Feature Extraction (CNN)		
			- Point voxel graph representation (GNN)		
Yang et al.	[119]	2022	- Patch sampling strategy	EXACT’09, BAS, and Private dataset	BAS: BD=89.01%, TD=92.71%, IoU=0.8738, Precision=0.9187
			- Channel-specific fuzzy attention		
Chen et al.	[131]	2022	- 3D U-Net	178 low-dose CT scans	Dice=0.81
			- Semi-supervised learning		
			- GAN		
Wu et al.	[133]	2022	- Two-stage framework	70 clinical chest CT scans	BD=90.83%, TD=87.59%, DSC=92.95%, FPR=0.03%
			- CNN		
			- A long-term slice propagation		
Wang et al.	[117]	2022	- Bronchiole sensitive loss function	EXACT’09, LIDC	LIDC: BD=83.3%, TD=90.4%, SEN=96.6%, DSC=94.2%, FPR=0.117
			- A human-vision-inspired iterative training strategy		
			- A semi-supervised learning framework		
Carmo et al.	[123]	2022	- Modified EfficientDet	Airway Tree Modelling challenge (ATM22)	Dice=93.49
Wang et al.	[132]	2023	- Modified- nnUNet- pseudo-label	EXACT’09, BAS, and Private dataset, ATM22	BAS: BD=96.4%, TD=91.4%, Precision=97.7%,
			- A tailored self-iterative learning scheme		Private dataset: BD=87.1%, TD=74.3%, Precision=97.8%
					EXACT’09: BD=86.5%, TD=87.1%, Precision=91.4%
					ATM22: BD=97.9%, TD=97.1%, DSC:92.8%, Precision=87.9%
Zhao et al.	[118]	2023	- Group Deep Dense Supervision	BAS	BAS: BD=90.5%, TD=95.8%, TPR=98.4%, FPR=0.134%
			- CNN		
Xie et al.	[127]	2023	- CNN + GNN	COPDGene	ACC=91.18%, TD=1.8
Wu et al.	[59]	2023	- Two stage framework	EXACT’09, BAS, ATM22	BAS: BD=92.4%, TD=94.9%, Precision=86.9%,
			- Contextual Transformer+CNN		EXACT’09: BD=81.4%, TD=79.6%, FPR=8.27%
					ATM22: BD=86.67%, TD=90.97%, DSC:94.06%, Precision=93.03%

AI quantifies airway alterations like wall thickness, diameter, or cross-sectional area. Such measurements facilitate objective assessment of airway disease severity and its progression over time. Nardelli et al. introduced a convolutional neural regressor trained with a generative model and Simulated and unsupervised generative adversarial network (SimGAN) to accurately characterize small pulmonary structures from CT images, overcoming the limitations of traditional methods. The validation results, both synthetic and in-vivo, showcase the promise of CNNs in providing accurate measurements of airway lumen, airway wall thickness, and vessel radius on chest CT images, potentially revolutionizing the diagnosis and treatment of pulmonary diseases [134].

#### AI enables airway changes aid in COPD identification

Beyond segmentation and quantification, AI’s prowess extends to classifying detected airway changes for diagnostic implications. It achieves this by: Pattern recognition: Algorithms detect specific airway patterns linked to COPD subtypes, enhancing diagnostic specificity [135, 136]. Integration with clinical data: AI models that merge imaging data with clinical parameters, such as spirometry, amplify the accuracy of COPD diagnosis and staging [137, 138]. Proactive predictions: Some models predict the risk of exacerbations or disease progression based on airway patterns, enabling clinicians to tailor management strategies proactively [138–141]. To encapsulate, AI’s role in airway analysis is transformative. By refining detection, segmentation, quantification, and classification processes, AI equips clinicians with invaluable insights, paving the way for personalized and effective COPD management.

### Vessel imaging analysis through AI

Vascular changes, both within the pulmonary system and potentially extending extrapulmonary, are intricately linked with COPD’s pathology. The nuanced visualization and interpretation of these vascular dynamics become paramount, and this is where the synergy between radiology and AI offers promising avenues.

#### Traditional vs. AI-enhanced visualization

Historically, radiological evaluations of pulmonary vessels were reliant on the expertise of radiologists to discern vessel caliber changes, pruning, or other abnormalities from CT scans. While effective, there were some limitations. Interpretations could vary between experts, especially in borderline cases or early disease stages. Some microvascular changes could escape the naked eye, potentially delaying interventions. AI’s integration bridges these gaps. Algorithms ensure a uniform approach, reducing discrepancies in vessel interpretation. AI tools can potentially identify sub-millimeter vascular changes, offering a more comprehensive view of vessel dynamics [111, 142].

#### Segmentation and quantification of pulmonary vessel

As shown in Table 6, the presented studies focus on the challenging task of vessel segmentation and artery-vein separation in CT images. Various approaches, including CNN methods [143–147], generative adversarial networks [53], and transformer-based networks [60], were proposed to address issues such as the separation of pulmonary arteries and veins, the synthesis of non-contrast and contrast-enhanced CT images, and the segmentation of intricate vessel structures. These methods leverage advanced architectures, such as 3D contextual transformers and channel-enhanced attention modules, to improve accuracy and efficiency. Evaluation on diverse datasets demonstrates the effectiveness of the proposed techniques, showcasing their potential applications in diagnosing and planning treatments for lung diseases. Additionally, the studies highlight the importance of addressing challenges such as limited annotated data and the need for robustness to noise in medical image segmentation tasks. Jimenez et al. presented a graph-cut methodology and a random forest pre-classifier for the segmentation of pulmonary artery-vein (AV) structures in CT images with an F1-score of 79.5% [148]. Cui et al. proposed an efficient 2.5D segmentation network from three orthogonal axes, achieving superior performance in pulmonary vessel segmentation with a Dice score of 0.9272 [149]. Gu et al. introduced two techniques for pulmonary vessel suppression, demonstrating improved nodule detection for early lung cancer diagnosis [145]. Nam et al. developed a deep learning-based pulmonary vessel segmentation algorithm (DLVS), showcasing high accuracy (AUC=0.977) and clinical relevance for assessing vascular remodeling in COPD patients [150]. Wu et al. addressed the limitations of FCN and U-Net in vessel segmentation, proposing the MSI-U-Net with attention mechanisms and achieving state-of-the-art results (DSC:0.7168, SEN:0.7234, and precision:0.7893) [151]. Li et al. presented a novel 3D vessel segmentation network guided by edge profiles, demonstrating superior performance with a DSC of 0.789, especially in scenarios with limited training data [152]. Wang et al. leveraged spatial registration for automatic pulmonary vessel segmentation in NCCT images, achieving high Dice of 0.856 [153]. Pan et al. tackled the challenges of artery-vein separation, introducing the MSIA-Net with multi-scale fusion blocks and achieving remarkable segmentation performance DSC:81.7%, precision:80.5%, and FPR:0.069% [154]. Pang et al. proposed synthesizers for mutual synthesis of NCCT and CECT images, showcasing their effectiveness in pulmonary vessel segmentation [53]. Wu et al. contributed a transformer-based network for vessel segmentation and artery-vein separation, demonstrating high accuracy and applicability in CT images [60]. These methods collectively advance the field, offering innovative solutions for accurate and efficient vessel segmentation in medical imaging.

**Table 6:** Summary of vessel segmentation and artery-vein separation methods by different teams

Team	Reference	Year	Task	Method Keypoints	Metrics
Nardelli et al.	[143]	2018	Artery-vein separation	CNN+Graph cut	ACC=93.6%
Xu et al.	[144]	2018	Vessel segmentation	Lung segmentation, fully convolutional network, region growing	ACC=0.998, SEN=0.894
Jimenez-Carretero et al.	[148]	2019	Artery-vein separation	A random forest, graph-cut	F1-score=79.5%
Cui et al.	[149]	2019	Vessel segmentation	2.5D CNN	Dice=0.9272, Precision=0.9310
Gu et al.	[145]	2019	vessel segmentation	Two cascade CNN	Dice=0.941, Jaccard index=0.890
Guo et al.	[146]	2020	Vessel segmentation	CNN	Dice=0.943
Nam et al.	[150]	2021	Vessel segmentation	A dual-source CT, 3D UNet	AUC=0.977
Qin et al.	[130]	2021	Artery-vein separation	Feature recalibration, attention distillation, CNN+graph-cut	ACC=97.2%, TPR=97.1%, FPR=0.015%, DSC=97.2%
Wu et al.	[151]	2022	Vessel segmentation	Multi-scale interactive CNN, attention mechanism	DSC=0.7168, SEN=0.7234, Precision=0.7893
Li et al.	[152]	2022	Vessel segmentation	CNN, LSTM	DSC=0.789, SEN=0.820, SPE=0.991, mIoU=0.819
Wang et al.	[153]	2023	Vessel segmentation	Contrast-enhanced (CE) CT labels, image registration, CNN	Dice=0.856
Pan et al.	[154]	2023	Artery-vein separation	Multi-scale CNN, centerline topology connectivity	ACC=98.0%, DSC=81.7%, Precision=80.5
					TPR=84.8%, FPR=0.069%
Pang et al.	[53]	2023	Vessel segmentation	Self-supervised learning, GAN, CNN	Dice=0.86
Wu et al.	[60]	2023	Vessel segmentation	CNN, contextual Transformer, double attention	Vessel segmentation:
					Dice=0.840 (CE CT)
					Dice= 0.867 (Non-contrast (NC) CT)
					Artery-vein separation:
					Dice=0.758 (CE CT)
					Dice=0.602 (NC CT)

Understanding vessel alterations requires not only meticulous segmentation but also quantification. AI quantifies vessel caliber, branching patterns, or other structural changes, offering metrics that are pivotal for COPD assessments and potential therapeutic responses. The quantitative analysis indicators of the vascular tree in patients with COPD provide significant insights about the disease’s progression and the patient’s overall health. These indicators include total blood volume (TBV), surface area, total volume of vessels with a cross-sectional area smaller than 5 mm^2^ (BV5), and BV5/TBV [155].

#### Extrapulmonary abnormalities & AI

While COPD predominantly affects the lungs, extrapulmonary manifestations, including cardiovascular implications, are notable [156, 157]. AI plays a role in the following aspects. Algorithms trained on diverse datasets can detect subtle extrapulmonary changes, offering insights into systemic COPD effects [58, 158]. AI not only visualizes but also interprets these manifestations in the context of COPD, potentially predicting risks like cardiac events or other systemic complications [159]. By merging pulmonary and extrapulmonary data, AI offers a holistic view of COPD’s impact, guiding comprehensive patient management [160, 161].

In conclusion, AI’s role in illuminating vessel dynamics, both pulmonary and extrapulmonary, revolutionizes our grasp of COPD’s vascular implications. This fusion of technology and radiology heralds a future where COPD management is not just reactive but proactive, underpinned by deep, data-driven insights.

## Clinical applicability and Challenges in clinical implementation of AI for COPD

### Clinical applicability of AI methods in COPD management

The clinical applicability of AI methods in COPD management is a critical consideration when assessing the real-world impact of these technologies. While numerous studies have demonstrated the promising performance of AI algorithms in various tasks, such as COPD identification and staging, emphysema region segmentation, and quantitative analysis, it is essential to evaluate their effectiveness and feasibility in actual clinical settings.

Several studies have shown that AI-assisted diagnosis can improve diagnostic accuracy and efficiency compared to traditional methods. For example, González et al. [66] demonstrated that their CNN-based approach achieved a high classification accuracy of 77.3% in identifying COPD patients from CT scans. Similarly, Xu et al. [70] reported an accuracy of 99.29% in identifying COPD using a modified AlexNet architecture. These results suggest that AI algorithms can potentially reduce misdiagnosis and improve the early detection of COPD, which is crucial for timely intervention and management.

Moreover, automatic measurements provided by AI algorithms can significantly enhance clinical workflow efficiency. Hasenstab et al. [74] developed an automated pipeline for quantifying emphysema and air trapping from CT scans, which showed strong correlations with manual measurements. Such automated tools can save substantial time for radiologists and pulmonologists, allowing them to focus on more complex cases and patient care.

However, it is important to acknowledge the limitations of current studies when considering their clinical applicability. Many of the cited works have relatively small sample sizes and lack long-term follow-up data, which may limit the generalizability of their findings. Supervised learning, particularly for segmentation tasks, necessitates accurately annotated images. Inconsistencies in labeling can mislead the model, compromising its efficacy. Deep learning models, especially, hunger for vast data volumes. Without enough examples, these models risk overfitting, limiting their generalizability. Balancing data access for model training and patient privacy is critical. Moreover, ethical considerations regarding data sourcing and utilization can’t be overlooked [162]. Furthermore, the integration of AI tools into existing clinical workflows and the interpretability of AI models remain significant challenges that need to be addressed for successful clinical adoption.

To fully assess the clinical applicability of AI methods in COPD management, future research should focus on conducting large-scale, prospective studies with diverse patient populations and long-term follow-up. Additionally, efforts should be made to develop more interpretable AI models and optimize their integration with clinical workflows. Real-world application cases and ongoing clinical trials, such as the COPDGene study [163], can provide valuable insights into the potential benefits and challenges of implementing AI tools in COPD care.

### Challenges in clinical implementation of AI methods

Harnessing the power of AI in the arena of COPD imaging offers boundless opportunities, but it also comes with inherent challenges. These challenges include the interpretability and explainability of AI models, integration into existing clinical workflows, and regulatory issues.

Interpretability and explainability are crucial for building trust and confidence in AI-assisted decision-making. Many AI models, particularly deep learning algorithms, operate as “black boxes”, making it difficult for clinicians to understand how the model arrived at a particular decision [164]. This lack of transparency can hinder the adoption of AI tools in clinical practice. To address this issue, researchers are developing methods to enhance the interpretability of AI models, such as Grad-CAM [165] and attention rollout [166]. These approaches aim to provide insights into the features and reasoning behind AI predictions, enabling clinicians to validate and trust the results.

Integrating AI tools into existing clinical workflows is another significant challenge. AI algorithms should seamlessly fit into the daily routines of healthcare professionals without causing disruptions or increasing workload [167]. This requires close collaboration between AI developers, radiologists, and pulmonologists to design user-friendly interfaces and workflows that align with clinical needs. Furthermore, the outputs of AI tools should be presented in a clear and actionable manner, allowing clinicians to easily incorporate the information into their decision-making process.

Regulatory issues pose another hurdle in the clinical implementation of AI methods. AI algorithms used in healthcare are subject to strict regulatory requirements to ensure patient safety and data privacy [168]. Compliance with these regulations often involves extensive validation, documentation, and monitoring, which can be time-consuming and resource-intensive. Moreover, the rapidly evolving nature of AI technologies presents challenges for regulatory bodies to keep pace with the latest developments and establish appropriate guidelines.

To overcome these challenges, a multi-faceted approach is necessary. Researchers should prioritize the development of interpretable and explainable AI models, engaging clinicians in the process to ensure clinical relevance. Collaborative efforts between AI developers and healthcare professionals are essential for designing intuitive and efficient workflows that integrate AI tools seamlessly. Additionally, regulatory bodies need to adapt and evolve their guidelines to keep up with the advancements in AI technologies while maintaining patient safety and data privacy standards.

## Future perspectives

### Emerging innovations: potential future directions and innovations in AI for COPD imaging

Enhanced AI algorithms: As AI research progresses, there’s a potential to develop algorithms that can better detect early COPD changes or subtypes not identifiable with current techniques. This implies a move beyond simple identification towards nuanced understanding, such as differentiating various pathophysiological processes or predicting the likelihood of exacerbations based on subtle imaging features. Adaptive learning systems: The concept of AI systems that evolve with each scan, constantly learning and refining their diagnostic abilities, could revolutionize timely and accurate disease detection and staging. 3D imaging reconstructions: With advancements in imaging modalities and AI-driven reconstructions, there’s a possibility of generating dynamic 3D models of the lungs. These models could offer real-time insights into airway dynamics, vascular changes, and tissue alterations, providing a depth of understanding previously unattainable. Beyond imaging - integrated diagnostic platforms: The future might see platforms that merge imaging data with physiological metrics, blood biomarkers, and even genomics. Such an integrated approach would provide a multi-dimensional perspective of COPD, facilitating precision medicine endeavors.

### Interdisciplinary collaborations: the fusion of AI with other scientific fields for comprehensive COPD management

AI and molecular biology: By integrating AI with molecular research, we could decipher intricate relationships between imaging phenotypes and molecular signatures. This could aid in identifying potential therapeutic targets or understanding the underpinnings of various COPD subtypes at a molecular level [169].

Neuroimaging and COPD: Emerging research hints at COPD’s neural implications. Collaborations between AI, pulmonology, and neuroimaging could uncover neural patterns associated with COPD, potentially opening avenues for novel interventions [170].

Environmental data and AI: By incorporating environmental data, AI models could predict COPD exacerbation risks based on localized air quality metrics, allergen levels, or other relevant factors. This integrative approach would encompass not just internal but also external factors influencing COPD dynamics [171].

Collaborations: The fusion of AI with other scientific fields for comprehensive COPD care. The potential of AI in enhancing the diagnostic and therapeutic landscape of COPD is undeniable. Yet, its full potential remains to be untapped. As we envisage the future, we recognize a trajectory marked by complex innovations and the melding of diverse disciplines to offer holistic and advanced COPD care.

Patient-centric platforms: The blend of AI with user experience (UX) design could lead to platforms that not only monitor patients but also educate and empower them. Such tools would make patients active participants in their care, promoting adherence and proactive health management [172].

Conclusively, the potential of AI in COPD care transcends mere imaging. Its nexus with diverse disciplines and the ensuing innovations could profoundly reshape COPD diagnostics, therapeutics, and patient engagement in the years to come.

## Conclusion

The application of AI, specifically machine learning and deep learning techniques, has shown significant potential in transforming the diagnosis and management of COPD. These technologies are providing unprecedented insights into aspects of the disease such as emphysema, airway dynamics, and vascular structures, which are critical for a holistic understanding and treatment of COPD. Despite the challenges posed by the complex ’black-box’ nature of AI algorithms and the need for robust model training, the future holds promise. The emergence of innovations in AI for COPD imaging and the potential for cross-disciplinary collaborations indicate a future where AI is not just an aid, but a leader in significant advancements in COPD care. However, to fully leverage AI’s potential, it’s imperative to create rich, meticulously annotated datasets that will help develop reliable and generalizable AI models. By doing so, we can ensure that AI contributes more effectively to the refinement of COPD patient care.

## Data Availability

No datasets were generated or analysed during the current study.

## Abbreviations

COPD: Chronic obstructive pulmonary disease
AI: Artificial intelligence
CT: Computed tomography
ML: Machine learning
DL: Deep learning
CNN: Convolutional neural network
GLCM: Gray level co-occurrence matrix
GLSZM: Gray level size zone matrix
GLRLM: Gray level run length matrix
NGTDM: Neighboring gray tone difference matrix
GLDM: Gray level dependence matrix
SVM: Support vector machine
MIL: Multiple instance learning
HRCT: High-resolution CT
CLE: Centrilobular emphysema
PLE: Panlobular emphysema
PSE: Paraseptal emphysema
HU: Hounsfield Unit
SR-Net: Slice-recovery network
ViT: Vision Transformer
BMI: Body mass index
FEV1: Forced expiratory volume in one second
FVC: Forced vital capacity
GOLD: Global initiative for chronic obstructive lung disease
SimGAN: Simulated and unsupervised generative adversarial network
AV: Artery-vein
TBV: Total blood volume
BV5: Total volume of vessels with a cross-sectional area smaller than 5 mm^2^
AUC: Area under the curve
ACC: Accuracy
DSC: Dice similarity coefficient
SEN: Sensitivity
SPE: Specificity
FPR: False positive rate
TPR: True Positive Rate
CE: Contrasted enhanced
NC: Non-contrasted
